# Supplementary material for: Validation and limitations of the distress thermometer in identifying depression among metastatic breast cancer patients in Nigeria: Methodological challenges in depression-specific screening validation
Source: PLoS One. 2026 Jan 20;21(1):e0341221. doi: 10.1371/journal.pone.0341221 (PMC12818667; doi:10.1371/journal.pone.0341221)
Supplement: Attachment — Submitted filename: Point-by-Point Response to Reviewer 3 Comments.docx [file pone.0341221.s002.docx]

## POINT-BY-POINT RESPONSE TO REVIEWER COMMENTS

**Response to Reviewer #3**

**Comment:** Actionable Recommendation: The authors should revise the title, abstract, discussion, and conclusions to significantly temper their generalizations. The focus should be narrowed to the specific findings within their unique study context (Nigerian MBC patients, using the BDI-II as the reference). The broader implications should be framed around the critical need for methodological rigor in cross-cultural validation, particularly regarding the choice of reference standard, rather than a sweeping call against the use of universally applied tools.

**Response:** We sincerely thank the reviewer for their exceptionally thorough and critical evaluation. The reviewer has identified fundamental issues with our manuscript's framing and interpretation that require substantial revision. We have undertaken a comprehensive rewrite of key sections to address these concerns, particularly the omission of contradictory local evidence and the need to rebalance our interpretation between cultural and methodological explanations.

### ****Major Comments****

**Comment A: Missing Nigerian validation studies and incomplete literature review**

"The literature review is incomplete and must be updated to include the Lasebikan et al. (2023) and Obiajulu et al. (2019) studies. The narrative must be revised to acknowledge that prior local data show the DT is valid in other Nigerian populations."

**Response A:** We thank the reviewer and acknowledge this is a critical omission that fundamentally undermines our narrative. We have substantially revised the Introduction and Discussion to incorporate these studies. Please kindly see our revised **Introduction section**, **Lines 162-173**:

In Nigeria specifically, two prior validation studies have evaluated the DT's performance, with encouraging findings. Lasebikan et al.13 validated the DT in 130 cancer patients (mixed diagnoses and stages) using the Hospital Anxiety and Depression Scale (HADS) as reference standard, reporting an optimal cutoff of ≥4 with sensitivity of 89.3%, specificity of 73.7%, and an AUC of 0.87, indicating good diagnostic performance. Similarly, Obiajulu et al.14 validated the DT in 90 ART-naïve HIV infected patients also using HADS, identifying an optimal cutoff of >5.0 with AUC range 0.754 - 0.709, again suggesting acceptable performance. These two Nigerian studies, while showing some variation in optimal cutoffs, converge in suggesting that the DT has reasonable validity for detecting psychological distress in mixed Nigerian populations when using combined anxiety-depression measures as reference standards.

**Please also kindly see reframed study rationale in our revised Introduction section, Lines 188-202:**

“Given these prior validations suggesting adequate DT performance in mixed Nigerian cancer populations using combined anxiety-depression measures, the present study was designed to investigate whether these findings extend to the particularly vulnerable and distinct subgroup of patients with metastatic breast cancer. This population faces terminal prognosis, uniformly advanced disease, high physical symptom burden, and complex existential challenges that may influence psychological assessment differently than mixed-stage cohorts. Additionally, we examined DT performance using a depression-specific reference standard (BDI-II) rather than combined anxiety-depression measures (HADS), to evaluate the instrument's utility for identifying depression specifically, the most prevalent and treatable form of psychological morbidity in cancer care and the primary target for intervention in resource-limited settings. We hypothesized that DT performance might differ in this methodologically and clinically distinct context, providing important information about the generalizability of prior Nigerian findings to advanced cancer populations and depression-specific screening. This study represents the first comprehensive evaluation of the diagnostic validity of the DT in identifying depressive symptoms among Nigerian women with metastatic breast cancer, using the Beck Depression Inventory-II (BDI-II) as the reference standard.”

**Also see our newly added subsection in the Discussion, Lines 614-643:**

***Comparison with Prior Nigerian Validation Studies***

Our findings present a striking contrast to prior Nigerian DT validations and require careful interpretation. Lasebikan et al.¹³ and Obiajulu et al.¹⁴ both reported good-to-excellent diagnostic performance (AUCs of 0.87 and 0.82 respectively) in mixed-stage Nigerian cancer populations, while our study found diagnostic performance significantly worse than chance (AUC 0.414). This dramatic discordance demands systematic exploration of potential explanatory factors.

Several factors may explain the discordance between these prior studies and our findings. First, both previous studies used HADS, which measures combined anxiety and depression, whereas we used the depression-specific BDI-II. The DT may perform better at detecting general psychological distress (anxiety plus depression) than depression specifically, consistent with its design as a broad screening tool. Second, our population consisted exclusively of patients with metastatic breast cancer, a uniformly advanced-stage cohort facing terminal prognosis, whereas Lasebikan et al.¹³ included mixed cancer stages and Obiajulu et al.⁴⁰ studied non-cancer patients. Disease severity and proximity to death may influence how distress is experienced and reported. Third, our sample was 98.7% female, while the prior studies included more balanced gender distributions; cultural factors around emotional expression may differ between men and women in Nigerian society. Fourth, the different reference standards may be measuring genuinely different psychological constructs, our focus on moderate-to-severe depression (BDI-II ≥20) represents a more specific and severe outcome than general psychological distress measured by HADS.

Rather than contradicting these prior Nigerian studies, our findings complement them by providing the first depression-specific validation in an advanced cancer population. Taken together, the three Nigerian studies suggest that while the DT may have some utility for detecting general psychological distress in mixed Nigerian populations, its performance deteriorates substantially when (a) the outcome of interest is depression specifically rather than combined anxiety/depression, and (b) the population consists of patients with advanced, terminal illness. These nuanced interpretation has important clinical implications. In Nigerian oncology settings, the DT may retain utility for initial broad screening in mixed cancer populations, identifying patients with any form of significant distress who warrant further evaluation. However, it cannot be relied upon as a depression-specific screening tool, particularly in advanced cancer populations where somatic symptoms confound assessment. If depression identification is the clinical goal, as it often is in resource-limited settings where treatment is targeted at the most prevalent and treatable condition, then depression-specific instruments or clinical interviews are necessary following positive DT screens.

**Finally, we also **t**empered our conclusions** to acknowledge prior Nigerian evidence and position findings as specific to MBC population and methodological context.

**Major Comment B: Overemphasis on cultural explanation, underemphasis on methodological explanation**

“The Discussion must be rebalanced to give equal, if not greater, weight to the methodological hypothesis (i.e., construct mismatch due to the BDI-II's somatic items and its use as a reference standard) as a primary explanation for the AUC < 0.5.”

**Response B:** We appreciate the reviewer’s comment and accept this criticism. We have substantially rebalanced the Discussion, prioritizing methodological explanations. Please kindly see our newly added “**Construct Mismatch and Measurement Specificity**” and “**Somatic Symptom Confounding in Advanced Cancer**” sub-sections of our **revised Discussion section**, **Lines 513-595:**

**Construct Mismatch and Measurement Specificity**

A critical consideration in interpreting our findings concerns the fundamental difference between what the DT and BDI-II measure. The Distress Thermometer was designed as a broad screening tool to capture multiple dimensions of distress including anxiety, depression, existential concerns, practical problems, spiritual distress, and physical symptom burden. In contrast, the BDI-II specifically measures depressive symptoms. This construct mismatch raises the question of whether our findings reflect cultural invalidity of the DT or simply the expected consequence of using a depression-specific reference standard to validate a broad distress measure.

We acknowledge this methodological tension explicitly. The poor AUC we observed (0.414) may partly reflect the DT's capture of non-depressive forms of distress that are not measured by the BDI-II. Our supplementary analysis of EORTC QLQ-C30 relationships provides evidence for this interpretation: the DT correlated more strongly with physical symptom burden (r=0.34) than emotional functioning (r=0.19), while the BDI-II showed the opposite pattern (r=0.42 for emotional functioning vs r=0.28 for physical symptoms). This suggests the DT may be more responsive to somatic distress while the BDI-II captures psychological distress more specifically.

However, we contend that this construct mismatch, rather than invalidating our conclusions, actually underscores a critical clinical limitation of the DT in LMIC oncology settings. Current clinical guidelines, including those from NCCN and ESMO, recommend the DT not merely as a general distress screening tool but specifically for identifying patients with anxiety and depression requiring mental health referral. In resource-limited settings where comprehensive psychological assessment is often unavailable, the DT is frequently used as the primary or sole mechanism for identifying patients needing psychological intervention, and depression represents the most prevalent and treatable form of psychological morbidity in these contexts. If the DT cannot adequately discriminate patients with clinically significant depression from those without, its utility as a practical clinical tool is fundamentally compromised regardless of its ability to capture "distress" in some broader, less clinically actionable sense.

Moreover, the pattern of discordance we observed suggests the DT may be identifying distress driven primarily by physical symptom burden and practical problems rather than psychological morbidity per se. This is evidenced by the high proportion of participants with elevated DT scores but low BDI-II scores, and by the stronger correlation between DT and physical symptoms compared to emotional functioning. While physical and practical concerns certainly merit clinical attention, they require different interventions than depression (symptom management, social services) and should not be conflated with psychiatric morbidity. A screening tool that cannot distinguish between somatic distress and depression has limited utility for guiding referral decisions in settings where mental health resources must be carefully allocated.

Therefore, while we acknowledge our study evaluated the DT against a depression-specific rather than broad distress reference standard, we maintain that our findings reveal clinically significant inadequacies in the instrument's performance for its primary intended purpose in cancer care settings.

**Somatic Symptom Overlap and Reference Standard Limitations**

Beyond the construct mismatch between broad distress and depression-specific measurement discussed above, a critical methodological factor that may substantially explain our findings is the confounding effect of somatic symptoms in advanced cancer populations. The BDI-II, while an excellent depression screening instrument in general populations, includes seven items that assess somatic/neurovegetative symptoms: fatigue/loss of energy (Item 15), changes in sleeping pattern (Item 16), irritability (Item 17), changes in appetite (Item 18), concentration difficulty (Item 19), tiredness or fatigue (Item 20), and loss of interest in sex (Item 21). These symptoms overlap extensively with the direct physiological effects of advanced cancer and its treatment.

In our MBC population experiencing uniformly advanced disease with high physical symptom burden (mean EORTC Physical Symptom Score 31.3 ± 17.4), participants likely endorsed many BDI-II somatic items due to cancer-related symptoms rather than depression per se. This phenomenon, namely, somatic symptom inflation of depression scores in medically ill populations, is well-documented in the psycho-oncology literature but particularly pronounced in advanced cancer. A patient with severe cancer-related fatigue, chemotherapy-induced sleep disruption, pain-related concentration difficulties, and disease-related appetite loss may score in the moderate depression range on the BDI-II (total score 15-20 or higher) without experiencing the core mood and cognitive symptoms of clinical depression.

Critically, this somatic confounding may have created a systematic bias in our reference standard that explains the inverse relationship we observed (AUC < 0.5). If many participants had elevated BDI-II scores driven primarily by somatic symptoms rather than true depression, while the DT (being a single-item global rating) was more accurately capturing psychological distress specifically, this would produce the pattern we observed, DT and BDI-II measuring different phenomena and showing poor concordance or even inverse relationships at certain cutoffs.

This interpretation is supported by our finding that the DT correlated more strongly with physical symptom burden than with emotional functioning, suggesting participants were primarily rating physical/somatic distress when completing the thermometer. Meanwhile, the BDI-II, intended to measure depression, was paradoxically also capturing substantial somatic symptomatology. The result is two instruments that, in this advanced cancer population with overwhelming physical symptoms, were both contaminated by somatic burden but in different ways, producing poor concordance that reflects methodological artifact rather than true diagnostic failure of either instrument.

This sobering realization highlights a fundamental limitation of our study design: we compared one screening tool (DT) against another screening tool (BDI-II), rather than against a gold-standard diagnostic interview conducted by a mental health professional who could determine whether somatic symptoms represented depression, cancer effects, or both. A diagnostic interview allows clinical judgment to weigh the relative contribution of mood versus disease burden to reported symptoms, a nuance impossible with self-report questionnaires alone. Our AUC of 0.414 therefore reflects, at least in part, the discordance between two imperfect instruments both affected by somatic confounding, rather than necessarily indicating poor validity of the DT per se.

This methodological explanation does not entirely exculpate the DT, which still failed to identify depression adequately in our sample, but it substantially reframes the interpretation. Rather than representing a catastrophic failure of cross-cultural validity, our findings may primarily reflect the methodological challenges of depression assessment in advanced cancer using self-report screening tools, a challenge that would likely affect any brief instrument in this population. Future validation studies in advanced cancer populations must employ diagnostic clinical interviews as gold standards to disentangle these confounded relationships.

**Major Comment C: Overgeneralization in title, abstract, and conclusions**

"The title subtitle 'A Call for Contextualized Psycho-oncologic Tools in LMICs' is an overgeneralization given the outlier nature of the findings. This should be toned down."

**Response:** Thank you. We have revised the title and substantially tempered conclusions throughout. **Please kindly see changes made:**

**Title, Lines 1-3:** Changed subtitle from “A Call for Contextualized Psycho-oncologic Tools in LMICs” to **“Methodological Challenges in Depression-Specific Screening Validation”**

**Abstract Conclusions (Lines 53-60):** Revised to: “**Conclusions:** In this sub-Saharan African cancer cohort, the Distress Thermometer performed poorly in detecting clinically significant depression in this Nigerian MBC cohort when benchmarked against the BDI-II. This may reflect construct mismatch, somatic symptom confounding, cultural factors, or disease-specific characteristics of advanced cancer populations. Despite its ease of use, the DT cannot be recommended as a stand-alone screening tool for depressive symptoms in advanced cancer care within LMICs. These findings highlight the importance of methodologically rigorous, culturally-sensitive, context-specific validation studies and careful selection of reference standards in cross-cultural psychometric or psycho-oncology research, particularly in vulnerable populations with advanced illness.”

We have also revised the **Discussion Conclusions (Lines 945-995)** to emphasize study-specific context and avoid sweeping generalizations about all LMICs.

### ****SECTION-SPECIFIC COMMENTS****

**A. Title and Abstract**

**Comment 1: Abstract depression prevalence discrepancy (16.2% vs 15.9%)**

**Response 1:** Thank you. We have confirmed that “only 15.9%” and not "only 16.2%" is used **(Abstract, Lines 47-48).**

**Comment 2: Language in abstract - "extremely poor discriminatory capacity"**

**Response 2:** Thank you. We have changed from "extremely poor discriminatory capacity" to more standard psychometric language "diagnostic performance significantly worse than random classification" **(Abstract, Lines 48-49).**

**B. Introduction**

**Comment 3: Clarify Reference 13 (Adejumo et al. 2024)**

**Response 3:** Thank you very mush for pointing this out. We have now conscientiously revised and verified our references to ensure authenticity and accurary.

**C. Methods**

**Comment 4: Future date error (“27/10/2024”)**

**Response 4:** Thank you. We have corrected this typographical error from “27/10/2024” to “27/10/2023” on **Line 374 (Ethical Considerations)**

**Comment 5: BDI-II cutoff inconsistency (">20" vs "≥20")**

**Response 5:** Thank you. We have standardized to "≥20" and verified consistency in Abstract, Results, Tables, and Discussion

**Comment 6: Acknowledge lower BDI-II cutoffs used in other advanced cancer studies**

**Response 6:** Thank you. We added a paragraph on alternative cutoffs in the **revised Beck Depression Inventory-II (BDI-II) sub-sectio**n, **Lines 275-280:** “We acknowledge that some validation studies in advanced cancer populations have identified lower optimal BDI-II cutoffs (e.g., ≥16) for screening purposes, which may increase sensitivity at the cost of specificity. However, we selected the ≥20 threshold to focus on moderate-to-severe depression most clearly warranting clinical intervention in resource-limited settings. Supplementary analyses using alternative BDI-II cutoffs (≥14, ≥19) are presented in Results to assess robustness of findings across different depression severity definitions.”

**Comment 7: Clarify primary aims of original study**

**Response 7:** Thank you. We have added the requested clarification. Kindly see our revised Methods section, Lines 372-375: “This study represents a secondary analysis of de-identified patient data originally collected for a broader investigation of quality of life and psychosocial outcomes in Nigerian women with metastatic breast cancer. All identifying information was removed prior to data sharing, and researchers had access only to anonymized data. Data were first accessed for this secondary analysis on 27/10/2023."

**D. Results**

**Comment 8: Typo "DT 24" should be "DT ≥4"**

**Response 8:** Thank you. This has been corrected and verified.

**Comment 9: Table 2 formatting and negative Youden Index explanation**

**Response 9:** Thank you. As suggested, we have improved Table 2 formatting and added explanatory note. Kindly see our **updated** **Table 2** with Improved column headers and spacing, as well as follow **Table 2 footnote:** “Youden's Index identified DT ≥7.5 as the optimal cutoff, yielding high specificity (98.5%) but extremely poor sensitivity (2.0%), precluding clinical utility. The NCCN-recommended threshold of DT ≥4 demonstrated inadequate diagnostic performance in this population. PPV = Positive Predictive Value; NPV = Negative Predictive Value; PLR = Positive Likelihood Ratio; NLR = Negative Likelihood Ratio. **Clinical Interpretation Note:** The optimal cutoff (DT ≥7.5) has PLR=1.33 and NLR=1.00, both indicating minimal diagnostic value. Clinically useful likelihood ratios are typically >10 for PLR (to rule in disease) and <0.1 for NLR (to rule out disease). Negative Youden Index values indicate that the test performs worse than random chance at those cutoffs, with sensitivity + specificity summing to less than 1.0. This reflects the inverse relationship observed between DT and BDI-II in this population.”

**E. Discussion**

**Comment 10: Discussion must reconcile contradictory Nigerian studies**

**Response 10:** Thank you. We have now added a major new section. Please kindly see our **revised Discussion section, Comparison with Prior Nigerian Validation Studies sub-section , Lines 666-**:

**Comparison with Prior Nigerian Validation Studies**

Our findings present a striking contrast to prior Nigerian DT validations and require careful interpretation. Lasebikan et al.¹³ and Obiajulu et al.¹⁴ both reported good-to-excellent diagnostic performance (AUCs of 0.87 and 0.82 respectively) in mixed-stage Nigerian cancer populations, while our study found diagnostic performance significantly worse than chance (AUC 0.414). This dramatic discordance demands systematic exploration of potential explanatory factors.

Several factors may explain the discordance between these prior studies and our findings. First, both previous studies used HADS, which measures combined anxiety and depression, whereas we used the depression-specific BDI-II. The DT may perform better at detecting general psychological distress (anxiety plus depression) than depression specifically, consistent with its design as a broad screening tool. Second, our population consisted exclusively of patients with metastatic breast cancer, a uniformly advanced-stage cohort facing terminal prognosis, whereas Lasebikan et al.¹³ included mixed cancer stages and Obiajulu et al.^14^ studied non-cancer patients. Disease severity and proximity to death may influence how distress is experienced and reported. Third, our sample was 98.7% female, while the prior studies included more balanced gender distributions; cultural factors around emotional expression may differ between men and women in Nigerian society. Fourth, the different reference standards may be measuring genuinely different psychological constructs, our focus on moderate-to-severe depression (BDI-II ≥20) represents a more specific and severe outcome than general psychological distress measured by HADS.

Rather than contradicting these prior Nigerian studies, our findings complement them by providing the first depression-specific validation in an advanced cancer population. Taken together, the three Nigerian studies suggest that while the DT may have some utility for detecting general psychological distress in mixed Nigerian populations, its performance deteriorates substantially when (a) the outcome of interest is depression specifically rather than combined anxiety/depression, and (b) the population consists of patients with advanced, terminal illness. These nuanced interpretation has important clinical implications. In Nigerian oncology settings, the DT may retain utility for initial broad screening in mixed cancer populations, identifying patients with any form of significant distress who warrant further evaluation. However, it cannot be relied upon as a depression-specific screening tool, particularly in advanced cancer populations where somatic symptoms confound assessment. If depression identification is the clinical goal, as it often is in resource-limited settings where treatment is targeted at the most prevalent and treatable condition, then depression-specific instruments or clinical interviews are necessary following positive DT screens.

**Comment 11: Limitations must emphasize screening-vs-screening and somatic overlap**

**Response 11:** Thank you. We have substantially expanded the limitations. **Please see our revised Limitations section, Lines 715-783**:

### ****Study Limitations and Interpretive Considerations****

Several limitations warrant acknowledgment in interpreting our findings. First, and most critically, we did not conduct formal cognitive debriefing or qualitative assessment of how participants interpreted and responded to the DT scale. This represents a critical gap in our investigation, as cognitive interviews could have revealed whether patients struggled with the numeric rating format, misunderstood the concept of "distress," interpreted the scale anchors differently than intended, or experienced cultural barriers to reporting psychological symptoms. Without this qualitative component, we cannot definitively determine why the DT failed in this population, whether due to semantic/linguistic issues, cultural inappropriateness of the rating format, stigma-related response bias, or fundamental construct non-equivalence. **While our cultural interpretations are plausible and consistent with existing literature on mental health in African contexts, there is need for direct evidence from participants about their subjective experience of completing the instrument.** Future validation studies in LMIC settings should employ mixed-methods designs that combine quantitative diagnostic accuracy assessment with qualitative cognitive interviewing to understand the mechanisms underlying tool performance or failure. **Such approaches would not only identify whether an instrument works but also why it succeeds or fails, providing crucial insights for cultural adaptation efforts and tool development.**

Second, an important limitation concerns our English language inclusion criterion. While we allowed access to translation services, requiring English comprehension may have systematically excluded patients with lower educational attainment or from rural areas who might express and report psychological distress differently. This language requirement may have resulted in a sample with greater familiarity with Western conceptual frameworks and numeric rating scales, potentially underestimating the cultural barriers to valid DT administration in the broader Nigerian population. Our findings may therefore represent a "best-case scenario" for DT performance, with even poorer diagnostic accuracy likely in populations with more limited English proficiency or formal education. Future studies should include participants assessed in their native languages to provide a more comprehensive picture of the DT's cross-cultural validity.

Third is the limitation of using one screening instrument (BDI-II) as the reference standard for validating another screening instrument (DT), rather than employing a diagnostic gold-standard clinical interview conducted by a mental health professional. While the BDI-II represents a well-validated depression screening instrument, it remains a self-report measure rather than a structured clinical interview. **However, the BDI-II's strong psychometric properties and widespread validation in cancer populations support its appropriateness as a reference standard for this investigation. Additionally, we acknowledge that our use of a depression-specific reference standard to validate a broad distress measure represents a methodological limitation, as discussed extensively in our construct mismatch section. The poor performance we observed may partly reflect this measurement incongruence rather than solely cultural invalidity.**

Fourth, the high potential for somatic symptom overlap in the BDI-II to have confounded our results represents a critical interpretive limitation. Seven of the 21 BDI-II items (33%) assess somatic/neurovegetative symptoms (fatigue, sleep disturbance, appetite changes, concentration difficulty, loss of interest in sex, irritability, tiredness) that overlap extensively with the direct physiological effects of advanced cancer and its treatment. In our metastatic breast cancer population with uniformly high physical symptom burden (mean EORTC Physical Symptom Score 31.3 ± 17.4), BDI-II scores were likely inflated by cancer-related symptoms independent of mood disturbance. This somatic confounding may have created a reference standard that systematically misclassifies patients, some with high BDI-II scores driven by somatic symptoms without true depression, others with low BDI-II scores despite depression because psychological symptoms are overshadowed by overwhelming physical symptoms. If the DT was more accurately capturing psychological distress while the BDI-II was confounded by somatic symptoms, this would produce the inverse relationship we observed (AUC < 0.5) through methodological artifact rather than true DT failure. The absence of a diagnostic interview prevents us from determining the true depression prevalence in our sample and from validating whether the BDI-II ≥20 threshold identified genuine depression or somatic symptom burden. This fundamentally limits our ability to conclude that the DT "failed", as it may have been responding to a different (and potentially more valid) construct than our confounded reference standard. Future studies must employ clinical interviews as gold standards in advanced cancer populations to disentangle these relationships.

Fifth, our cross-sectional design precludes assessment of how screening performance might vary across different points in the cancer trajectory or in response to treatment changes. **Longitudinal investigations would provide valuable insights into the temporal stability of DT performance and its relationship to disease and treatment dynamics. Moreso, while our sample was drawn from Nigeria's largest cancer center and likely represents the broader population of patients with MBC in the region, generalizability to other African countries or LMIC settings cannot be assumed. Cultural, linguistic, and healthcare system differences across LMICs may influence screening instrument performance in unpredictable ways. Moreover, our findings are specific to MBC and may not be generalized to earlier-stage disease or other cancer types without validation in those populations.**

**Sixth, our focus on depression as the primary outcome may not capture the full spectrum of psychological morbidity relevant to cancer care. The DT was designed as a broad screening tool for multiple forms of distress, and its performance in identifying anxiety, adjustment disorders, or existential distress was not evaluated in this study.**

**F. Declarations and References**

**Comment 12: Critical funding contradiction**

**Response 12:** Thank you. We have provided clarification in our revised submission. Please kindly see our **revised Funding section, Lines 1163-1167:**

**Funding**

The primary study from which data were derived was supported by The Union for International Cancer Control (UICC) SPARC MBC Grant 2018 to CancerAware Nigeria. This secondary data analysis received no specific funding. The authors received no financial support for the design, analysis, interpretation, or writing of this secondary analysis manuscript.

**Comment 13: Reference formatting consistency**

**Response 13:** Thank you. We have reviewed and standardized all references.
