# Supplementary material for: Validation and limitations of the distress thermometer in identifying depression among metastatic breast cancer patients in Nigeria: Methodological challenges in depression-specific screening validation
Source: PLoS One. 2026 Jan 20;21(1):e0341221. doi: 10.1371/journal.pone.0341221 (PMC12818667; doi:10.1371/journal.pone.0341221)
Supplement: Attachment — Submitted filename: Point-by-Point Response to Reviewer #3 Comments.docx [file pone.0341221.s003.docx]

## POINT-BY-POINT RESPONSE TO REVIEWER COMMENTS

**Response to Reviewer #3**

We are deeply grateful to Reviewer #3 for their exceptionally thorough, critical, and intellectually rigorous review of our manuscript. The reviewer has identified fundamental conceptual and framing issues that required substantial revision throughout the manuscript. We acknowledge that many of the reviewer's critiques are well-founded and represent important improvements to the scientific rigor and interpretive balance of our work. We have undertaken extensive revisions to address these concerns, fundamentally reframing our findings to emphasize methodological explanations over cultural interpretations, acknowledging the inherent limitations of our reference standard choice, and substantially tempering our conclusions. All changes are marked with tracked changes in the revised manuscript. We believe these revisions have transformed this into a more scientifically sound, appropriately caveated, and intellectually honest contribution to the literature.

**1. Abstract and Title**

**Comment:** "Misleading Conclusion: The abstract concludes the DT 'cannot be recommended as a stand-alone screening tool for depressive symptoms.' This is too strong. A more accurate conclusion would be: 'The DT should not be used to screen specifically for depression in this population, as it measures a broader construct. Its utility for identifying general distress remains unclear due to methodological limitations in this study.'

Omission of Key Limitation: The abstract does not mention the critical limitation of using a depression-specific reference standard to validate a general distress tool, nor the confounding effect of somatic symptoms on the BDI-II in an advanced cancer population.".

**Response:** We sincerely thank the reviewer and completely agree with this critique. Thus, we have substantially revised the Abstract conclusion to be more precise and appropriately caveated. **Please kindly see our revised Abstract conculusion, Lines 48-58**:

**Conclusions:** In this sub-Saharan African cancer cohort, the Distress Thermometer performed poorly in detecting clinically significant depression in this Nigerian MBC cohort when benchmarked against the BDI-II. This may reflect construct mismatch, somatic symptom confounding, cultural factors, or disease-specific characteristics of advanced cancer populations. The DT should not be used as a depression-specific screening tool in advanced cancer populations in LMICs, though its utility for identifying general distress remains unclear given the methodological limitations of this study. The poor concordance observed likely reflects construct mismatch, somatic symptom confounding in the reference standard, and the fundamental challenges of depression assessment in advanced cancer populations using self-report instruments. These findings underscore the critical need for appropriate reference standards (structured clinical interviews) and highlight methodological considerations in validating psychosocial screening tools across different constructs and cultural contexts.

### 2. ****Introduction****

**Comment:** "Unsubstantiated Hypothesis: The hypothesis that 'DT performance might differ in this methodologically... distinct context' is vague. A more precise hypothesis would have been: 'We hypothesize that the DT will demonstrate poor diagnostic accuracy for depression specifically, due to construct mismatch and somatic confounding, despite its previously reported utility for general distress.'"

**Response:** We thank the reviewer and agree that our original hypothesis was imprecise and failed to articulate the expected mechanisms underlying poor performance. We have completely rewritten the hypothesis to specify both the expected finding and the mechanistic explanations. Please kindly see our revised **Introduction section**, **Lines 196-207**:

We hypothesized that the DT would demonstrate suboptimal diagnostic accuracy for identifying depression specifically (as distinct from general distress) in this population, due to: (1) construct mismatch between the broad, multidimensional distress captured by the DT and the specific depressive symptoms assessed by the BDI-II; and (2) potential somatic symptom confounding given the high physical symptom burden characteristic of metastatic cancer, which may inflate BDI-II scores independent of mood disturbance. This investigation provides important information about the DT's performance for a specific clinical application (depression screening) in advanced cancer populations, complementing prior Nigerian validation studies that examined general psychological distress using combined anxiety-depression measures. This study represents the first comprehensive evaluation of the diagnostic validity of the DT in identifying depressive symptoms among Nigerian women with metastatic breast cancer, using the Beck Depression Inventory-II (BDI-II) as the reference standard.

3. **Methods**

**Comment:** "Fatal Flaw in Reference Standard Selection: The choice of BDI-II as the reference standard is the study's critical weakness. The authors provide a justification (Lines 276-292), but it is inadequate.

Construct Mismatch: They acknowledge the DT measures 'broad distress' and the BDI-II measures 'depression,' yet they proceed with the comparison. This is like validating a thermometer (measures temperature) against a barometer (measures pressure) and concluding the thermometer has failed when the readings don't correlate.

Somatic Confounding: The BDI-II contains multiple items (e.g., fatigue, sleep changes, appetite loss) that are intrinsic to advanced cancer and its treatment. In a population with a high physical symptom burden (mean EORTC physical symptom score 31.3), the BDI-II score is almost certainly inflated by physical illness rather than mood. The authors later realize this (Lines 762-766) but it should have been a primary reason not to use it as a gold standard.

Lack of a True Gold Standard: The only scientifically sound reference standard for a diagnostic accuracy study of depression in medically ill patients is a structured clinical interview (e.g., SCID, MINI) conducted by a trained mental health professional, which can clinically distinguish depressive symptoms from somatic symptoms of cancer."

**Response:** We appreciate the reviewer’s comment and accept this critique. We have substantially revised the Methods section to (i) Explicitly acknowledge the construct mismatch upfront rather than burying it, (ii) Frame our study as examining a specific clinical application (depression screening) rather than validating the DT's full intended purpose, (iii) Acknowledge that structured clinical interviews represent the true gold standard, and (iv) Emphasize that our findings reflect methodological challenges rather than definitive cross-cultural invalidity. Please kindly see the“**Beck Depression Inventory-II (BDI-II)**” sub-sections of our **revised Methods section**, **Lines 267-311:**

**Beck Depression Inventory-II (BDI-II)**

The Beck Depression Inventory-II represents a well-validated, 21-item self-report instrument designed to assess the severity of depressive symptoms over the preceding two-week period.²¹ Individual items are scored on a 4-point scale (0-3), yielding total scores ranging from 0 to 63. The instrument demonstrates excellent psychometric properties, with reported Cronbach's alpha coefficients typically exceeding 0.90 in both clinical and non-clinical populations.

For this study, we employed a cutoff score of ≥20 to define clinically significant depressive symptoms, corresponding to moderate-to-severe depression severity. This threshold aligns with established guidelines for clinical practice and research applications, represents the level of symptom severity most likely to benefit from professional intervention, and has been validated in previous oncology studies.²²⁻²³

We acknowledge that some validation studies in advanced cancer populations have identified lower optimal BDI-II cutoffs (e.g., ≥16) for screening purposes, which may increase sensitivity at the cost of specificity. However, we selected the ≥20 threshold to focus on moderate-to-severe depression most clearly warranting clinical intervention in resource-limited settings. Supplementary analyses using alternative BDI-II cutoffs (≥14, ≥19) are presented in Results to assess robustness of findings across different depression severity definitions.

We selected the BDI-II as our reference standard with full acknowledgment of the inherent construct mismatch in using a depression-specific instrument to validate a broad distress screening tool. This represents a significant methodological limitation, as discussed extensively in our limitations section. However, we pursued this design for specific pragmatic and clinical reasons, recognizing that our findings speak to the DT's performance for one particular clinical application (identifying patients with depression) rather than validating its full intended scope of measuring general distress. We made this methodological choice for several reasons. First, depression represents the most prevalent and clinically significant form of psychological morbidity in advanced cancer populations, and is the primary target for psychological intervention in most LMIC oncology settings where mental health resources are scarce. Second, while other DT validation studies have used broader measures such as the BSI-18, HADS total score, or DSM-IV interviews capturing multiple forms of distress, the BDI-II offers superior feasibility for implementation in resource-limited environments, requiring no specialized training for administration. Third, despite the DT's intended broad scope, clinical guidelines frequently recommend it for identifying patients with depression requiring referral for mental health services, making depression-specific validation clinically relevant. We explicitly acknowledge that our focus on depression as the outcome represents a narrower evaluation than the DT's full intended purpose, and that poor performance for depression detection does not necessarily indicate failure to capture other forms of distress. However, if the DT cannot adequately identify depression, the most common and treatable form of psychological morbidity in cancer, its clinical utility remains limited regardless of performance for other distress dimensions.

Nonetheless, we acknowledge that the ideal reference standard for depression validation in medically ill populations is a structured clinical interview (e.g., SCID, MINI) conducted by trained mental health professionals, which can distinguish depressive symptoms from somatic symptoms of cancer. The use of a self-report measure (BDI-II) as our reference standard, particularly one containing substantial somatic content, represents a significant limitation that may have contributed to the observed poor concordance. Our findings should be interpreted as reflecting the challenges of depression screening using brief self-report tools in advanced cancer populations, rather than definitively establishing the DT's cross-cultural invalidity.

4. **Results**

**Comment 1:** "Misinterpretation of AUC < 0.5: An AUC of 0.414, significantly below 0.5, is extraordinary. It doesn't just mean 'worse than random'; it suggests a systematic inverse relationship. The most plausible explanation is not that the DT is 'catastrophically' broken, but that the two instruments are measuring different, inversely related constructs in this context. The DT may be capturing physical and practical distress, while the BDI-II is contaminated by somatic items, creating this perverse result. The authors' later discussion of somatic confounding (Lines 762-766) is the correct interpretation, but it is buried and contradicts the main narrative."

**Response:** We thank the reviewer for this excellent and important point. We have revised the Results section to (i) Reframe the AUC < 0.5 finding as suggesting systematic measurement of different constructs rather than DT "failure", (ii) Reference the methodological explanation early rather than burying it in Discussion, and (iii) Remove language suggesting catastrophic failure. **Please kindly see changes made in Lines 447-456:**

**ROC Curve Analysis**

The ROC curve analysis revealed extremely poor diagnostic performance of the DT in identifying participants with clinically significant depressive symptoms (Figure 2). The AUC was 0.414 (95% CI: 0.326–0.503), significantly below the threshold for acceptable diagnostic accuracy (p < 0.001 for comparison with AUC = 0.5). This poor concordance between the DT and BDI-II in identifying participants with clinically significant depressive symptoms likely reflects that these instruments measure different psychological constructs in this population. As discussed in our limitations section, this pattern is consistent with the DT capturing primarily physical and practical distress while the BDI-II, containing substantial somatic content, is confounded by cancer-related symptoms in this advanced-stage population.

**Comment 2:** "Discordance Framing: The discordance (47% distressed vs. 16% depressed) is framed as a failure of the DT. An alternative, equally valid interpretation is that it highlights that "distress" in this population is driven more by physical and practical problems than by the specific psychological construct of depression."

**Response:** We thank the reviewer for this comment. We have also revised this portion of the Results section framing observed discordance as consistent with measuring different phenomena, interpreting the discordance pattern as distress driven by physical/practical versus mood problem, and acknowledging both instruments likely confounded by somatic symptom. **Please kindly see changes made in Lines 422-431:**

**Discordance Between Measures**

Substantial discordance was observed between DT-defined distress and BDI-II-defined depression, consistent with these instruments measuring different psychological phenomena. Of the 145 participants with DT ≥4, only 33 (22.8%) also met criteria for clinically significant depression (BDI-II ≥20). Conversely, 17 of the 49 participants (34.7%) with BDI-II ≥20 scored below the DT threshold for distress. This substantial lack of concordance (κ = 0.12, indicating poor agreement) suggests that these instruments are measuring distinct, albeit related, psychological constructs. It is also plausible that distress in this population is driven more by physical and practical problems (captured by the DT) than by the specific psychological construct of depression (assessed by the BDI-II), though both instruments are likely confounded by somatic symptoms in this advanced cancer population.

5. ****Discuss**ion**

**Comment 1: "Primary Explanation is Methodological, Not Cultural: The discussion leads with cultural and construct validity explanations. However, the most parsimonious explanation for the extreme results (AUC < 0.5) is the methodological artifact of using a somatically-confounded, depression-specific instrument to validate a general distress tool. The cultural explanations are plausible for a modestly reduced AUC (e.g., 0.6), but not for a complete diagnostic breakdown.**

**Somatic Confounding as an Afterthought: The critical 'Somatic Symptom Overlap' section (Lines 549-593) should be the centerpiece of the discussion's limitations. It fundamentally reframes the entire study. The admission that 'our findings may primarily reflect the methodological challenges of depression assessment in advanced cancer... a challenge that would likely affect any brief instrument in this population' (Lines 600-603) is a crucial caveat that undermines the broader claims about the DT's cross-cultural invalidity."**

**Response 1:** We sincerely appreciate the reviewer's important critique. Addressing the reviewer’s concern that our original discussion structure prioritized cultural interpretations when the evidence more strongly supports methodological explanations, we have undertaken a MAJOR RESTRUCTURING of the Discussion section (i) Moving the "Somatic Symptom Overlap" section to IMMEDIATELY follow "Principal Findings" (making it the PRIMARY explanation), (ii) Moving "Construct Mismatch" section to second position, (iii) Repositioning cultural factors as CONTRIBUTING rather than PRIMARY explanations, (iv) Adding explicit signposting that methodological factors are the most parsimonious explanation, and (v) Toning down all claims about cross-cultural invalidity throughout. Please kindly see our **revised Discussion section.**

Please kindly see our **revised Discussion section, Somatic Symptom Overlap and Reference Standard Limitations sub-section , Lines 538-587**:

**Somatic Symptom Overlap and Reference Standard Limitations**

Beyond the construct mismatch between broad distress and depression-specific measurement discussed above, a critical methodological factor that may substantially explain our findings is the confounding effect of somatic symptoms in advanced cancer populations. The most parsimonious explanation for our findings, including the extraordinary AUC below 0.5, is the fundamental methodological artifact created by using a somatically-confounded, depression-specific self-report instrument to validate a broad distress screening tool in an advanced cancer population. This methodological issue likely contributes more substantially to the observed poor concordance than cultural factors or cross-cultural invalidity of the DT itself. The BDI-II, while an excellent depression screening instrument in general populations, contains seven items (33% of total) that assess somatic/neurovegetative symptoms: fatigue/loss of energy (Item 15), changes in sleeping pattern (Item 16), irritability (Item 17), changes in appetite (Item 18), concentration difficulty (Item 19), tiredness or fatigue (Item 20), and loss of interest in sex (Item 21). These symptoms overlap extensively with the direct physiological effects of advanced cancer and its treatment.

In our MBC population experiencing uniformly advanced disease with high physical symptom burden (mean EORTC Physical Symptom Score 31.3 ± 17.4), participants likely endorsed many BDI-II somatic items due to cancer-related symptoms rather than depression per se. This phenomenon, namely, somatic symptom inflation of depression scores in medically ill populations, is well-documented in the psycho-oncology literature but particularly pronounced in advanced cancer. A patient with severe cancer-related fatigue, chemotherapy-induced sleep disruption, pain-related concentration difficulties, and disease-related appetite loss may score in the moderate depression range on the BDI-II (total score 15-20 or higher) without experiencing the core mood and cognitive symptoms of clinical depression.

Critically, this somatic confounding may have created a systematic bias in our reference standard that explains the inverse relationship we observed (AUC < 0.5). If many participants had elevated BDI-II scores driven primarily by somatic symptoms rather than true depression, while the DT (being a single-item global rating) was more accurately capturing psychological distress specifically, this would produce the pattern we observed, DT and BDI-II measuring different phenomena and showing poor concordance or even inverse relationships at certain cutoffs.

This interpretation is supported by our finding that the DT correlated more strongly with physical symptom burden than with emotional functioning, suggesting participants were primarily rating physical/somatic distress when completing the thermometer. Meanwhile, the BDI-II, intended to measure depression, was paradoxically also capturing substantial somatic symptomatology. The result is two instruments that, in this advanced cancer population with overwhelming physical symptoms, were both contaminated by somatic burden but in different ways, producing poor concordance that reflects methodological artifact rather than true diagnostic failure of either instrument.

This sobering realization highlights a fundamental limitation of our study design: we compared one screening tool (DT) against another screening tool (BDI-II), rather than against a gold-standard diagnostic interview conducted by a mental health professional who could determine whether somatic symptoms represented depression, cancer effects, or both. A diagnostic interview allows clinical judgment to weigh the relative contribution of mood versus disease burden to reported symptoms, a nuance impossible with self-report questionnaires alone. Our AUC of 0.414 therefore reflects, at least in part, the discordance between two imperfect instruments both affected by somatic confounding, rather than necessarily indicating poor validity of the DT per se.

This methodological explanation does not entirely exculpate the DT, which still failed to identify depression adequately in our sample, but it substantially reframes the interpretation. Rather than representing a catastrophic failure of cross-cultural validity, our findings may primarily reflect the methodological challenges of depression assessment in advanced cancer using self-report screening tools, a challenge that would likely affect any brief instrument in this population. Future validation studies in advanced cancer populations must employ diagnostic clinical interviews as gold standards to disentangle these confounded relationships.

**Comment 2: "Internal Contradiction: In 'Construct Mismatch and Measurement Specificity' (Lines 512-525), the authors correctly argue that the DT's clinical utility is limited if it can't identify depression. However, this is a clinical opinion, not a psychometric finding. The study did not validate the DT for its intended purpose (broad distress); it invalidated it for a purpose it was not solely designed for (depression-specific screening).**

**Overstated Conclusions: Language like 'systematic failure' and 'clinically meaningless' is too strong given the methodological context. The DT may be failing at the specific task the authors set for it, but that does not mean it has no utility."**

**Response 2:** We thank the reviewer. We agree that our language may have been too strong and failed to distinguish between "the DT failed at the specific task we tested" versus "the DT has failed as a tool." We have now systematically revised language throughout the Discussion to (i) Remove or soften "systematic failure," "clinically meaningless," "catastrophic," "profound failure", (ii) Add the qualifiers "for depression-specific screening," "in this methodological context," "given our reference standard choice", and (iii) Distinguish between clinical application and psychometric properties. Please kindly see our **revised Discussion section.**

**Some examples of our LANGUAGE REVISIONS include:**

Location 1: Principal Findings (**Lines 523-524**)

ORIGINAL: "reveals profoundly concerning findings regarding its diagnostic utility"

REVISED: "reveals concerning findings regarding its utility for depression-specific screening"

Location 2: Principal Findings (**Lines 524-526**)

ORIGINAL: "Our results demonstrate not merely suboptimal performance, but rather systematic failure of the DT to identify clinically significant depressive symptoms"

REVISED: "Our results demonstrate poor concordance between the DT and BDI-II for identifying depressive symptoms, likely reflecting methodological limitations rather than systematic failure of the DT as a screening instrument"

Location 3: Optimal Cutoff Discussion (**Lines 533-534**)

ORIGINAL: "demonstrated sensitivity so poor (2.0%) as to render the tool clinically meaningless for depression screening"

REVISED: "demonstrated very poor sensitivity (2.0%), indicating inadequate performance for depression-specific screening in this methodological context"

Location 4: Conclusions (**Lines 999-1001**)

ORIGINAL: "This study provides compelling evidence that the Distress Thermometer, despite its widespread international endorsement and ease of use, demonstrates extremely poor diagnostic performance in identifying clinically significant depressive symptoms"

REVISED: "This study demonstrates that the Distress Thermometer, despite its widespread international endorsement and ease of use, is not suitable for depression-specific screening in advanced cancer populations when validated against depression-focused self-report measures"

All instances of overly strong language have been systematically softened throughout our manuscript.

6. **Conclusions & Implications**

**Comment: "Unwarranted Generalization: The conclusion that the DT 'cannot be recommended' is too broad. A more nuanced conclusion would be: 'The DT should not be used as a depression-specific screener in advanced cancer populations in LMICs. Its role in identifying general distress requires further validation using appropriate reference standards that account for somatic symptom burden.'**

**Ignoring the 'Why': The study's design makes it impossible to determine why the DT performed poorly—whether due to cultural factors, the construct mismatch, somatic confounding, or a combination. Therefore, policy implications about tool abandonment are premature. The implication should be to improve validation study methodology."**

**Response:** We appreciate the reviewer’s comment. Consistent with the reviewer’s comment, we have substantially revised the Conclusions section (i) Specifying recommendations apply to depression-specific screening, not general use, (ii) Acknowledging that general distress utility remains unclear, (iii) Emphasizing need for better validation methodology rather than tool abandonment, and (iv) Acknowledging uncertainty about causal mechanisms. Please kindly see our **revised Conclusions section, Lines 998-1061:**

## ****Conclusions****

This study demonstrates that the Distress Thermometer, despite its widespread international endorsement and ease of use, is not suitable for depression-specific screening in advanced cancer populations when validated against depression-focused self-report measures. **With an AUC of 0.414, the instrument performed significantly worse than random classification in this unique methodological and clinical context, suggesting systematic failure rather than merely suboptimal performance. However, the interpretation of these findings requires careful consideration of multiple explanatory factors that extend beyond simple cross-cultural invalidity**

Our results diverge markedly from two prior Nigerian validations showing good DT performance in mixed-stage cancer and non-cancer populations using combined anxiety-depression measures (HADS) as reference standards. This discordance likely reflects methodological differences, specifically, our use of a depression-specific rather than broad distress reference standard, our focus on uniformly advanced-stage disease with high somatic symptom burden, and the fundamental limitation of validating one screening tool against another rather than against diagnostic clinical interviews. The substantial somatic symptom overlap in both instruments (DT capturing physical distress, BDI-II including somatic depression items) may have created confounding that explains much of the poor concordance observed, particularly in our metastatic population with overwhelming physical symptoms.

This study demonstrates that the Distress Thermometer should not be used as a depression-specific screening tool in advanced cancer populations in LMICs, though its utility for identifying general distress remains unclear and requires validation using appropriate reference standards. The poor concordance with the BDI-II observed in this study most likely reflects the methodological challenges of validating a broad distress measure against a depression-specific, somatically-confounded self-report instrument in an advanced cancer population, rather than definitively establishing the DT's cross-cultural invalidity.

Our study's design, using one self-report instrument to validate another, beclouds the possibility of determining with certainty whether the poor performance reflects construct mismatch, somatic symptom confounding, cultural factors, or some combination thereof. However, the most parsimonious interpretation, given the extraordinary AUC < 0.5 and the substantial somatic overlap in our reference standard, is that methodological artifacts may have substantially contributed to the observed findings. These results underscore the critical importance of using structured clinical interviews as gold standards for depression validation in medically ill populations and highlight the need for methodologically rigorous validation studies that appropriately match screening tools with reference standards measuring the same construct.

Our results demonstrate that effective psychosocial screening in oncology requires more than simply translating and implementing validated instruments from high-income countries. **Instead, meaningful progress demands recognition of cultural, methodological, and contextual factors that influence how psychological distress is experienced, expressed, and reported across diverse populations.**

**This investigation represents a crucial first step toward developing evidence-based, culturally appropriate approaches to psychological assessment in sub-Saharan African cancer populations. However, substantial additional research and health system development will be required to translate these insights into improved clinical care. The broader significance of this work lies not in definitively proving DT failure, but in demonstrating the critical importance of rigorous, context-specific validation before implementing screening tools in new populations or clinical contexts. Our outlier findings relative to prior Nigerian studies underscore how methodological choices (reference standard selection, population characteristics, outcome definition) fundamentally shape validation results and clinical conclusions.**

The implications extend beyond Nigeria to the broader global cancer community, highlighting the urgent need for local validation of imported health technologies and the development of contextually appropriate alternatives. **Only through such efforts can we ensure that psychosocial oncology advances reach the patients who need them most, regardless of geographic location or cultural background.**

Our findings represent an important contribution to understanding the complexities of cross-cultural psychometric validation, particularly in vulnerable populations with advanced illness. However, we emphasize that our findings are specific to depression screening in metastatic breast cancer using a particular reference standard, and cannot be generalized to all forms of distress, all cancer stages, all LMIC settings, or all screening contexts. The field requires additional studies employing diagnostic interviews, cognitive debriefing, and careful attention to somatic confounding to build a more comprehensive evidence base for psychosocial assessment in diverse global oncology populations.

The complexity of psychological assessment in cancer care, particularly across cultural contexts and disease stages, demands nuanced interpretation and cumulative evidence rather than definitive proclamations based on individual investigations. We call upon international cancer organizations, funding agencies, researchers, and healthcare providers to prioritize the development and validation of culturally sensitive psychosocial screening approaches for LMIC cancer populations. **The failure to address these disparities perpetuates global inequities in comprehensive cancer care and undermines efforts to achieve universal health coverage and cancer control.**

**We believe that addressing the Reviewer's critiques have fundamentally helped to improve our manuscript. The extensively revised manuscript now (a) leads with methodological explanations as primary factors, (b) frames findings as reflecting a specific clinical application (depression screening) not comprehensive validation, (c) acknowledges profound uncertainty about causal mechanisms, (d) uses appropriately caveated language throughout, and (e) emphasizes need for better methodology rather than tool abandonment**

**We believe this represents a much more scientifically rigorous, intellectually honest, and appropriately humble contribution to the literature. We are sincerely grateful for the reviewer's investment in improving this work.**
